# Regulation of Prepro-NeuropeptideW/B and Its Receptor in the Heart of ZDF Rats: An Animal Model of Type II DM

**DOI:** 10.3390/ijms232315219

**Published:** 2022-12-02

**Authors:** Shashank Pandey, Dagmar Jarkovska, Zdenek Tuma, Tereza Smrhova, Magdalena Chottova Dvorakova

**Affiliations:** 1 Biomedical Center, Faculty of Medicine in Pilsen, Charles University, 32300 Pilsen, Czech Republic; 2 Department of Pharmacology and Toxicology, Faculty of Medicine in Pilsen, Charles University, 32300 Pilsen, Czech Republic; 3 Department of Physiology, Faculty of Medicine in Pilsen, Charles University, 32300 Pilsen, Czech Republic

**Keywords:** neuropeptide B, neuropeptide W, RT-qPCR, ZDF rat, western blot, laser capture microdissection, NPBWR1, calcium transients, contraction, cardiomyocytes

## Abstract

Neuropeptide B (NPB) and neuropeptide W (NPW) are neuropeptides, which constitute NPB/W signaling systems together with G-protein coupled receptors NPBWR1. The location and function of NPB/W signaling systems have been predominantly detected and mapped within the CNS, including their role in the modulation of inflammatory pain, neuroendocrine functions, and autonomic nervous systems. The aim of the study is to investigate the impact of diabetes on the neuropeptide B/W signaling system in different heart compartments and neurons which innervates it. In the RT-qPCR analysis, we observed the upregulation of mRNA for preproNPB in RV, for preproNPW in LA, and for NPBWR1 in DRG in diabetic rats. On the contrary, the expression of mRNA for NPBWR1 was downregulated in LV in diabetic rats. In the WB analysis, significant downregulation of NPBWR1 in LV (0.54-fold, *p* = 0.046) in diabetic rats was observed at the proteomic level. The presence of NPBWR1 was also confirmed in a dissected LCM section of cardiomyocytes and coronary arteries. The positive inotropic effect of NPW described on the diabetic cardiomyocytes in vitro could point to a possible therapeutic target for compensation of the contractile dysfunction in the diabetic heart. In conclusion, the NPB/W signaling system is involved in the regulation of heart functions and long-term diabetes leads to changes in the expression of individual members of this signaling system differently in each cardiac compartment, which is related to the different morphology and function of these cardiac chambers.

## 1. Introduction

Cardiovascular autonomic neuropathy (CAN) belongs to a group of serious complications of diabetes, which exert a significant negative effect on the quality of life and survival of people with diabetes mellitus (DM) [1]. CAN is the result of damage to nerve fibers that innervate the heart muscle and coronary arteries. It affects both afferent and efferent components of parasympathetic and sympathetic innervation of the heart and has been detected in the early stages of both types of DM [2]. The activity of the sympathetic part of cardiac innervation is increased in individuals with DM, while the activity of the parasympathetic nervous system in the heart of these individuals decreases [2]. In the diabetic heart, in addition to the signaling systems of classical neurotransmitters, the signaling systems of various neuropeptides are also more or less affected including, e.g., neuropeptide Y [3,4,5], vasoactive intestinal polypeptide [6], substance P [4,7], and calcitonin gene-related peptide [8,9,10], suggesting impairment of whole intracardiac nervous system. This impairment contributes to the cardiac dysfunction and heart failure associated with DM [11]. Heart failure is the most common cardiovascular complication of type 2 DM and a major cause of premature mortality [12].

Neuropeptide B (NPB) and neuropeptide W (NPW) are recently discovered neuropeptides, which are structurally and functionally related endogenous peptides. They constitute NPB/W signaling systems together with receptors through which they act on target tissues. These are G-protein coupled receptors GPR7 (also called NPBWR1) expressed in humans, rats, and mice, and GPR8 (also called NPBWR2), which has been detected only in humans [13]. NPB/W signaling systems have an important role in regulating different physiological processes including feeding behavior, energy homeostasis, neuroendocrine function, and modulating inflammatory pain, but they also have a potential role in regulating stress responses [13]. Serum level of both NPB, as well as NPW, has been shown to be altered in patients with DM, and these neuropeptides could be considered serum predictors for the prognosis of nutritional status in type 1 DM in pediatric patients [14]. Zucker diabetic fatty (ZDF) rats, an animal model of type 2 DM, exerts higher NPW plasma level in comparison to controls [15]. These results suggest a potential role of NPB/W signaling systems in the pathophysiology of diabetes.

The expression and distribution of individual members of this signaling system were recently demonstrated in the heart of a rat [16]. The aim of this study is to determine whether and to what extent this signaling system is affected by type 2 DM, and what is the functional impact of this effect on the heart. Additionally, upper thoracic dorsal root ganglia (DRG) and stellate ganglia were included in the evaluation in order to examine the effect of DM on the expression of the NPB/W signaling system members in sympathetic and sensory nerve cell bodies innervating the heart. Various methodological approaches have been used, including RT-qPCR, western blot, immunofluorescence, and functional measurement of the isolated cardiomyocytes.

## 2. Results

### 2.1. Animals

Non-fasting serum glucose levels were 30.21 ± 3.01 mmol/L in diabetic (ZDF) rats (*n* = 16), and were 7.04 ± 1.39 mmol/L in control (lean Zucker) rats (*n* = 16; data given as mean ± SD). The body weight of the animals in both experimental groups were not significantly different at the time of sacrificing (Table 1).

### 2.2. Expressions of NPB, NPW, and NPBWR1 mRNAs

Messenger RNAs coding for the NPB, NPW, as well as NPBWR1, were detected in each heart chamber (LA, RA, LV, and RV) of the controls, as well as the ZDF animals. Overall, it can be stated that the Cq values in all tested tissues for all tested genes ranged from 29–35, indicating a very low level of gene expression.

**Figure 1:**
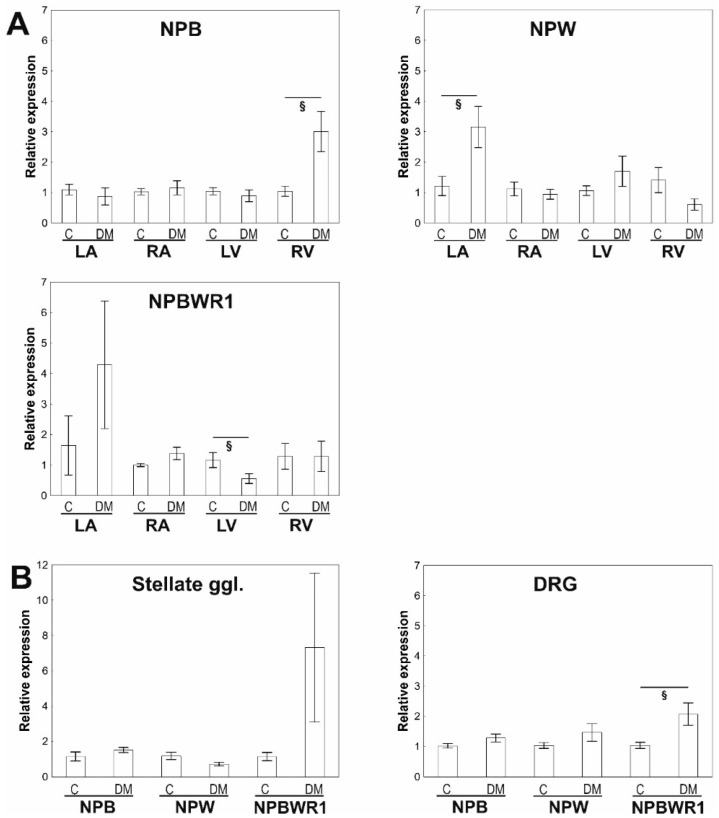
Quantitative RT-PCR analyses of NPB, NPW, and NPBWR1 mRNAs in separate heart compartments, dorsal root ganglia (DRG), and stellate ganglia of the control rats and Zucker diabetic fatty (ZDF) rats with type 2 diabetes mellitus. (**A**)—Comparison of expression of studied genes between the control and diabetic animals within separate heart compartments: left atrium (LA), right atrium (RA), left ventricle (LV), and right ventricle (RV). Data are presented as relative expression ± SEM. Control values of the appropriate heart compartments were used as comparators and were settled as 1. Statistically significant differences between the heart compartments are shown graphically. *n* = 5–8 in each group. (**B**)—Comparison of expression of studied genes between control and diabetic animals within dorsal root ganglia (DRG) and stellate ganglia. Statistically significant differences are shown graphically. *n* = 5–8 in each group. ^§^ *p* < 0.005 (Mann–Whitney test).

In the heart, long-term DM affects the expression of mRNA for preproNPB only within RV, where statistically significant upregulation was detected (3.0-fold, *p* = 0.018). Moreover, NPW mRNA expression was upregulated in the LA (3.2-fold, *p* = 0.022). Contrary, the expression of NPBWR1 mRNA was downregulated in the LV (0.56-fold, *p* = 0.029) (Figure 1A). 

In the DRG and stellate ganglia, the expression of mRNAs for preproNPB, NPW, and NPBWR1 was detected at similar or even lower levels than in heart tissues. DM caused an increase in the expression of mRNA for NPBWR1 in DRG, as well as SCG, but only in DRG was the overexpression statistically significant. Expressions of preproNPB and NPW mRNAs were not significantly different in DRG and stellate ganglia of the control and diabetic animals (Figure 1B).

**Figure 2:**
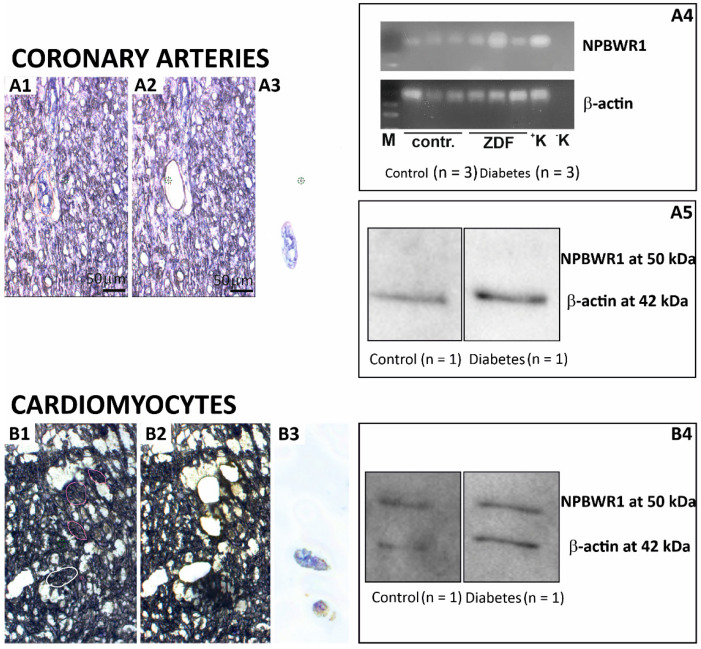
LCM dissection of coronary arteries (**A1**–**A4**) and cardiomyocytes (**B1**–**B4**) from the left ventricle of the rat heart: Hematoxylin-stained heart tissue of 13 µm thickness on laser microscope at 20× resolution. Appropriate areas were marked for cutting through the laser within sections (**A1**,**B1**). Tissue sections after precise laser cutting and collecting the cut pieces (**A2**,**B2**). Captured pieces collected on the lid of microtubes (**A3**,**B3**). PCR analysis of the LCM section: detection of expression of β-actin and NPBWR1 genes (**A4**). Western blot analysis of the LCM section (0.5 mm²/lane) with anti-NPBWR1 (1:2000) and anti-ACTB (1:250); an immunogenic band of ACTB at 42 kDa was identified in smooth muscles of LV (**A5**); immunogenic bands of ACTB at 42 kDa and NPBWR1 at 50 kDa were identified in cardiomyocytes of LV (**B4**).

In samples of coronary arteries from LV obtained by LCM, the expression of mRNA for NPBWR1 was detected in both the control and ZDF rats in very small amounts. The Cq values were above 35, suggesting a very limited expression of this gene within the vessels (Figure 2A).

### 2.3. Expressions of NPB, NPW, and NPBWR1 Proteins

For the WB analysis, we have only selected those diabetic tissues which were showing significant differences in the expression of mRNA levels in genomic studies. Selected tissues were RV (for preproNPB), LA (for preproNPW), LV (for NPBWR1), LA (for NPBWR1), and DRG (for NPBWR1). 

The protein expression was analyzed for NPBWR1 in LA (*n* = 6), preproNPW in LA (*n* = 5), NPBWR1 in LV (*n* =5), preproNPB in RV (*n* = 7), and preproNPB in DRG (*n* = 7). We have detected single immunogenic bands of NPBWR1 and preproNPB at 50 kDa and 25 kDa, respectively. Two immunogenic bands were observed for preproNPW at 35 and 26 kDa. However, we have considered only 26 kDa bands for preproNPW analysis because they showed 90% inhibition in pre-adsorption studies (Pandey and Chottova Dvorakova, 2020). 

**Figure 3:**
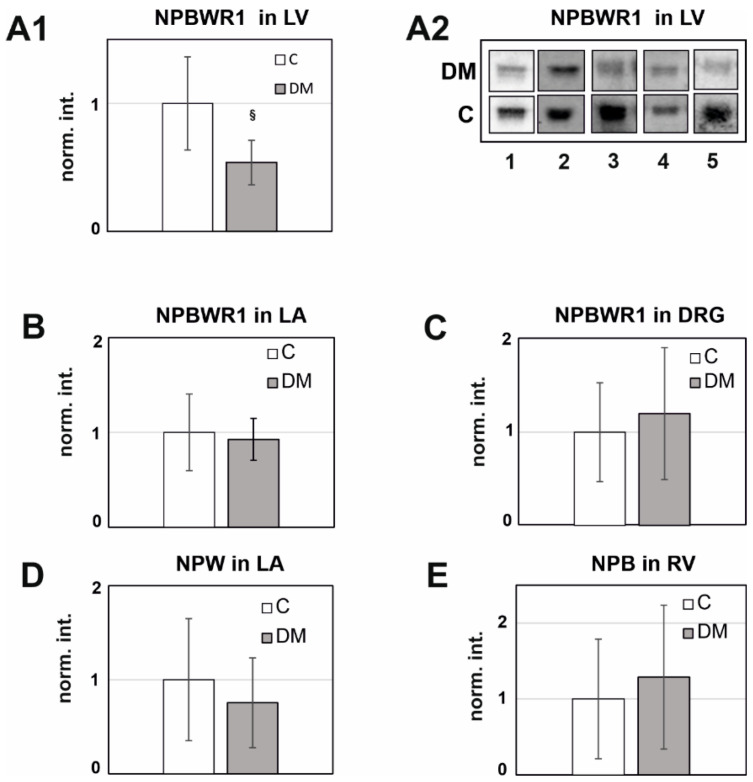
The quantitative WB analysis of rat heart tissues and DRG. Western blot (WB) analyses were performed using commercially available antibodies against NPB (1:200), NPW (1:1000), and NPBWR1 (1:2000) on selected tissues showing significant differences in qPCR analysis. Twenty-five µg of protein from the separated heart chamber was subjected to SDS-PAGE 12.5% and used for the WB analysis. (**A1**): LV from the diabetic rat (*n* = 5) versus the control rat (*n* = 5) using anti-NPBWR1 (1:2000) was used in WB. The signal ratio DM/C was 0.53 (*p* = 0.046). (**A2**): Immunogenic bands of NPBWR1 from LV of the diabetic heart versus the control heart. (**B**): LA from the diabetic rat (*n* = 6) versus the control rat (*n* = 6) using anti-NPBWR1 (1:2000). The signal ratio DM/C was 0.93 (*p* = 0.71). (**C**): DRG from the diabetic rat (*n* = 7) versus the control rat (*n* = 7) using anti-NPBWR1 (1:2000). The signal ratio DM/C was 1.21 (*p* = 0.55). (**D**): LA from the diabetic rat (*n* = 5) versus the control rat (*n* = 5) using anti-NPW (1:1000). The signal ratio DM/C was 0.86 (*p* = 0.74). (**E**): RV from the diabetic rat (*n* = 7) versus the control rat (*n* = 7) using anti-NPB (1:200). The signal ratio DM/C was 1.29 (*p* = 0.55). The final expression ratio in the diabetic animals was calculated by a *t*-test, where the average diabetic rat tissue signal was divided by the average control rat tissue signal in each group. All the data are expressed as the mean ± standard deviation. The values of *p* < 0.05 were considered statistically significant. ^§^ *p* < 0.005 (Mann–Whitney test).

The long-term effect of DM was also observed in the proteomic level and NPBWR1 was significantly underexpressed in diabetic LV (0.54-fold, *p* = 0.046). On the contrary, preproNPB in RV and NPBWR1 in DRG were overexpressed in diabetic rats, but the difference was not statistically significant (Figure 3).

The presence of NPBWR1 was further confirmed in dissected cardiomyocytes by the WB analysis. We identified an immunogenic band of NPBWR1 at 50 kDa in cardiomyocytes dissected from LV of the diabetic (*n* = 1) and control (*n* = 1) rats. However, no band for NPBWR1 was observed in the WB analysis of dissected coronary arteries from LV of the diabetic (*n* = 1) and control (*n* = 1) rats (Figure 2).

### 2.4. Immunofluorescence

**Figure 4:**
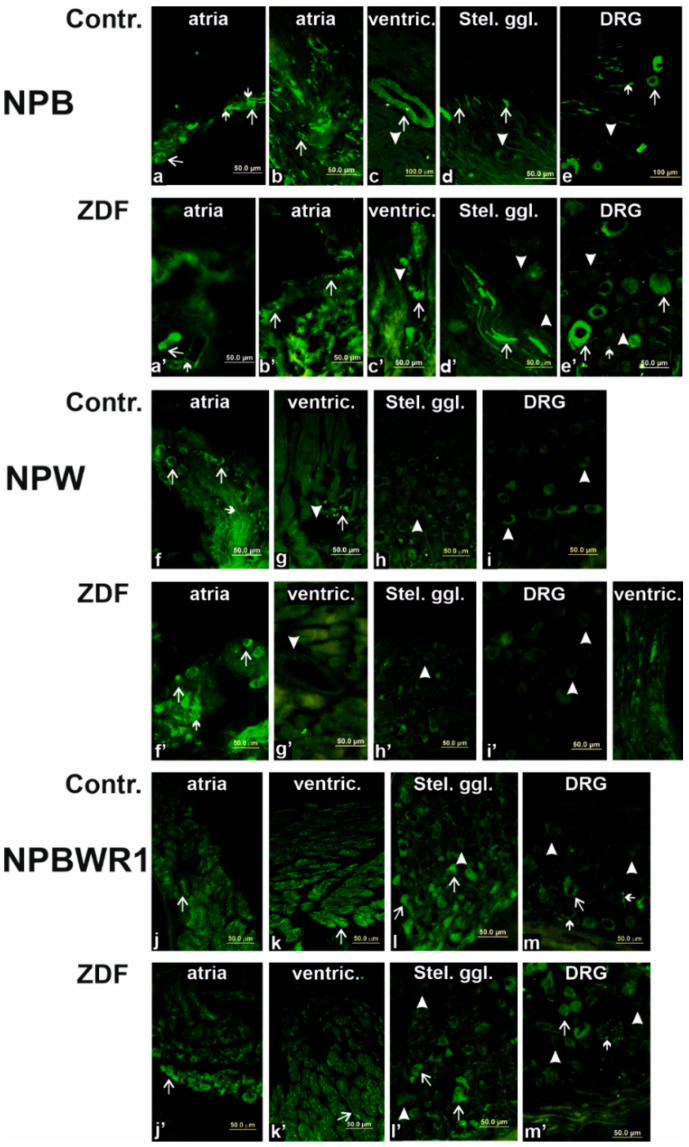
Immunofluorescence for neuropeptide B (NPB), neuropeptide W (NPW), and receptor 1 for NPW and NPB designed as NPBWR1 in the rat heart (atria and ventricles), stellate ganglia, and dorsal root ganglia (DRG) of the control and Zucker diabetic fatty (ZDF) rats. Within atria (**a**,**a’**,**b**,**b’**), antiserum against NPB exerted specific immunoreactivity (IR) in neuronal bodies and nerve fibers (some of them marked by arrows) in between cardiomyocytes and near-to-heart ganglia. Within ventricles (**c**,**c’**), smooth muscle cells of coronary arteries exerted specific IR. Stellate ganglia (**d**,**d’**): NPB-IR nerve fibers (some of them marked by arrows) and NPB-negative nerve cell bodies (arrowheads). DRG (**e**,**e’**): NPB-IR some nerve cell bodies (arrows) and nerve fibers (small arrows), some nerve cell bodies NPB-negative (arrowheads). Within atria (**f**,**f’**), NPW-IR nerve fibers (small arrows) and nerve cell bodies (arrows) are visible. Within ventricles (**g**,**g’**), just the very rare NPW-IR nerve fibers (arrows) are present. Smooth muscle cells of coronary vessels (**g**,**g’**) does not exert IR with NPW antiserum (arrowheads). No specific NPW-IR is visible in stellate ganglia (**h**,**h’**) and DRG (**i**,**i’**). Within atria (**j**,**j’**) and ventricles (**k**,**k’**), NPBWR1 antiserum show IR in the cardiomyocytes cell membrane (some marked by arrows). Stellate ganglia (**l**,**l’**): some nerve cell bodies are NPBWR1-IR (arrows) but some are negative (arrowheads). DRG (**m**,**m’**): specific NPBWR1-IR is visible in some nerve cell bodies (arrows) and some nerve fibers (small arrows). Other nerve cells are NPBWR1-negative (some of them marked by arrowheads).

Within the heart, specific NPB immunoreactivity (IR) was detected in nerve fibers densely innervating intracardiac ganglia (Figure 4a,a’). Some nerve cell bodies of intracardiac ganglia were also anti-NPB immunoreactive (Figure 4a,a’). IR nerve fibers were present also in between cardiomyocytes of atria (Figure 4b,b’) and, to a much lesser extent, ventricles. Additionally, smooth muscle cells of coronary vessels exerted specific IR with antiserum against NPB (Figure 4c,c’). 

Specific NPW-IR was detected in some nerve cell bodies and nerve fibers in the vicinity of neurons in intracardiac ganglia (Figure 4a,a’). There were no visible immunoreactive nerve fibers in between ventricular cardiomyocytes. Non-homogenous specific NPBWR1-IR was observed on the surface of atrial, as well as ventricular cardiomyocytes (Figure 4j,j’,k,k’). No difference in NPB, NPW, and NPBWR1 immunofluorescence distribution was detected between the control and diabetic heart tissues. 

Within the stellate ganglia, nerve cell bodies exert specific NPBWR1-IR but not IR with antibodies against NPB and/or NPW (Figure 4d,d’,h,h’,l,l’). Additionally, NPB-IR nerve fibers were detectable mainly on the surface of the ganglia (Figure 4d,d’). 

Some, but not all neurons, exerted NPB-IR within DRG (Figure 4e,e’). Additionally, NPB-IR nerve fibers were detected in between DRG neurons (Figure 4e,e’). Contrarily, no specific labeling was present with NPW antiserum (Figure 4h,h’). NPBWR1-IR was visible on some DRG neurons, as well as a few nerve fibers in the vicinity of the neurons.

### 2.5. Functional Measurement of Isolated Cardiomyocytes

**Figure 5:**
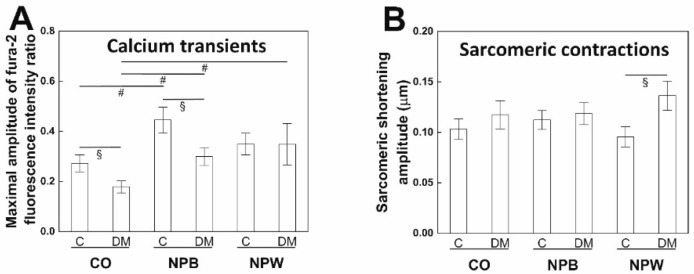
Functional measurement was performed on cardiomyocytes isolated from the left ventricle of the control rats and Zucker diabetic fatty rats with type 2 diabetes mellitus. (**A**)—Comparison of calcium transient amplitudes between the control and diabetic animals in the control solution, the solution containing NPB (0.1 μM), and the solution containing NPW (0.1 μM). *n* = 12–20 in each group. (**B**)—Comparison of sarcomeric shortening amplitudes between the control and diabetic animals in the control solution, the solution containing NPB (0.1 μM), and the solution containing NPW (0.1 μM). *n* = 16–23 in each group. Data are presented as mean ± SEM. Values of *p* < 0.05 were considered statistically significant. ^§^—significantly different from the control animals (Mann–Whitney test). #—significantly different from the control solution (Mann–Whitney test).

In the control solution, the maximal amplitude of the fura-2 fluorescence intensity ratio (Figure 5A) was significantly smaller in the diabetic animals. Both neuropeptides NPB and NPW applied in vitro caused an increase in this parameter in the diabetic animals. In the control animals, the increase was statistically significant only in the solution with NPB. In other words, the difference between the control and diabetic group was preserved only in the presence of NPB. After the application of NPW, the difference between the two animal groups was suppressed. Moreover, the diastolic decrease in intracellular calcium concentration was faster in diabetic animals in the solution with NPW (timed to 90% of the fura-2 fluorescence intensity decay 262 ± 26 ms in the control group versus 167 ± 13 ms in the diabetic group).

There was no significant difference in the sarcomeric shortening amplitude between the control and diabetic animals observed in the control solution and the solution containing NPB (Figure 5B). On the other hand, we saw a higher amplitude of sarcomeric contractions in diabetic animals when NPW was applied. In the presence of NPW, the relaxation was faster in diabetic animals (timed to 90% relaxation 305 ± 25 ms in the control rats versus 229 ± 16 ms in the diabetic rats). No similar effect was observed in the control solution or the solution containing NPB. 

## 3. Discussion

The NPB/W signaling system is expressed in many parts of the central nervous system and studies have suggested the involvement of the NPB/W signaling system in the regulation of several physiological processes. Moreover, the presence of the NPB/W signaling system in non-neuronal tissues has also been confirmed by some authors, including lymphatic tissue, organs of the gastrointestinal tract, some glands, and the heart [13,16]. 

The aim of this study is to investigate the genomic and proteomic expression and distribution of the NPB/W signaling system in the control and diabetic rat hearts and autonomic ganglia supplying the heart. Given that (1) there are functional and morphological differences between the individual cardiac compartments, and (2) that cardiac innervation also shows various morphological and functional differences between the right and left heart compartments [17], we included the individual cardiac compartments separately in the analysis. ZDF rats were used in the study, which is considered to be one of the most popular animal models of type 2 diabetes mellitus [18]. The impact of diabetes on the expression of members of the NPB/W signaling system in the sympathetic, sensory, as well as intracardiac innervation of the heart, was also examined. Long-term diabetes causes a reduction in the number of nerve fibers in the left ventricle but the same effect was not observed in the right ventricle [19], indicating a different effect of diabetes on each heart compartment.

Sympathetic postganglionic neurons innervating the heart are localized within the cervical and upper thoracic paravertebral ganglia, including also stellate ganglia [17]. Hyperglycemia, a hallmark sign of diabetes mellitus, is involved in the pathophysiology of autonomic neuropathy, including also CAN, in which all parts of cardiac innervation are affected [2]. 

In our previous study, we identified and confirmed the presence of mRNAs in all three members of the NPB/W signaling system in each heart chamber (LA, RA, LV, and RV) of non-diabetic rats, which was further validated by the WB analysis [16]. Since the NPB was found only in the fibers and not in the bodies of stellate ganglia neurons, we can assume that the NPB-IR fibers originate from the preganglionic neurons located in the intermediolateral nuclei of the spinal cord. NPB coming from the preganglionic neuron can affect/regulate the activity of stellate ganglia neurons through NPBWR1, the presence of which on the surface of these neurons has been demonstrated in our experiments. The involvement of NPW in the transmission of information in sympathetic cardiac innervation is uncertain. Although we have demonstrated the expression of the NPW gene in stellate ganglia, we were unable to detect the presence of NPW through the WB analysis and immunofluorescence. Moreover, we did not observe any impact of diabetes on the NPB/W signaling system in sympathetic cardiac innervation at the genomic and proteomic levels. These results suggest that the NPB/W signaling system is unlikely to be involved in impaired information transfer in the sympathetic portion of the autonomic innervation of the heart associated with diabetes mellitus. The influence of this signaling system on the sympathetic regulation of cardiac activity has been demonstrated in the past. In 2007, Yu with co-workers demonstrated that intracerebroventricular but not intravascular administration of NPW30 can increase heart rate and blood pressure [20]. From the above, it is clear that the sympathetic regulation of cardiac activity through NPW occurs at the level of the cardiovascular center in the central nervous system, which in turn increases the activity of the heart through the activation of cardio-stimulating sympathetic fibers. Still, the adverse impact of long-term diabetes on the regulatory system has yet to be established.

Parasympathetic postganglionic neurons innervate the heart from ganglia in the atrial wall, especially in the left atrium [21]. Both neuropeptides, NPB and NPW, are expressed in some of these neurons, as has been already demonstrated [16]. These experiments indicated that a higher number of cardiac neurons express NPB than NPW. LCM combined with RT-PCR and IF experiments have shown that NPW is present in the cell bodies and fibers of neurons in the heart, while NPB occurs in smooth muscle cells of the coronary circulation. Prolonged diabetes led to a statistically significant increase in left atrial NPW expression at the genomic level. We were unable to demonstrate a change in NPW expression in the left atrium at the proteomic level. 

The effect of NPW, as well as NPB, on target cells is mediated by NPBWR1 in rats [13]. In the heart, this receptor is present on the surface of cardiomyocytes, as evidenced by the results of IF experiments. Our previous results show that the relative expression of this receptor is much higher in heart ventricles than in the atria [16]. Furthermore, the presence of NPBWR1 on the surface of coronary smooth muscle cells can be expected, while this receptor was detected in the vascular muscle cells of the mesenteric artery. There the receptor was localized together with L-type calcium channels, which are known to regulate vascular myogenic tone [22]. Further experiments demonstrated that activation of these receptors by the application of NPW-23 has led to a contraction of the smooth muscle cells [22]. The effect of NPB has not been tested yet. However, the presence of this receptor in the smooth muscle of the coronary circulation was not confirmed by the results of the WB analysis, as well as IF experiments. Messenger RNA for NPBWR1 was detected in left ventricular coronary arteries but its level was very low. Thus, the expression level could be lower than the sensitivity of the WB and IF methodology. The presence of NPBWR1 receptors would also make sense here in view of our further finding that coronary smooth muscle cells contain NPB, which could in a paracrine/autocrine manner affect the tone of coronary vessels. Based on these results, it is more probable that the statistically significant decrease in NPBWR1 expression in the left ventricle is not due to a change in the expression of this receptor in the coronary circulation vessels, but rather in the cardiomyocytes. We also confirmed the expression of this receptor in cardiomyocytes by WB and IF. 

The left and right ventricles are morphologically and functionally different and, therefore, their different responses to pathological conditions can be expected. Cardiomyocytes from the left and right ventricles are different from several points of view, e.g., structural, metabolic, and electrophysiological characteristics [23,24]. Several pathological processes, including diabetes, lead to a change in the properties of cardiomyocytes [25,26]. A recently published work describes the different effects of DM on the cardiomyocyte morphology of individual ventricles [27]. This could explain why the difference in the expression of NPBWR1 caused by diabetes was detected just in LV. 

The functional impact of the decreased NPWR1 expression in the LV of diabetic animals was investigated at the cellular level. The calcium transient amplitude was decreased in the cardiomyocytes from diabetic animals, similar to other animal models, db/db type 2 diabetic mice [28], and streptozotocin-induced type 1 diabetic rats [29]. The calcium transient reduction was not observed in studies on type 2 diabetic ZDF rats [30,31] which were performed on 10 and 14 weeks, i.e., younger animals than in the presented study, however. The diminished calcium transient did not cause a significant change in the amplitude of sarcomeric contraction in diabetic cardiomyocytes which followed previous findings in ZDF rats [30,31]. A possible explanation could be provided by an increased myofilament calcium sensitivity serving as a compensatory mechanism in ZDF rats [32]. NPB in vitro application positively affected calcium transients from both animal groups with no effect on the contractility. NPW administration led to increased calcium transients resulting in increased contractility; however, only in the cells isolated from diabetic animals. Previously, the NPW was proven to stimulate voltage-dependent L-type Ca^2+^ (I_CaL_) channels in pancreatic beta cells and vascular smooth muscle cells [22,33], and thus the enhanced I_CaL_ channel function could be a reason for our findings. The intracellular signaling pathway in cardiomyocytes which is affected by the NPB and NPW remains to be elucidated. In other cell types, the NPB and NPW were shown to act via PKA and PKC signaling pathways intracellularly [34,35]. The effect of NPW observed in diabetic animals and not the control ones are in contradiction to the lower expression of NPWR1 in the diabetic left ventricles and suggest that NPWR1 is not the only receptor on the cardiomyocyte membrane binding NPW. The positive inotropic effect of NPW on diabetic cardiac cells could also represent a possible therapeutic target for compensation of contractile dysfunction in the diabetic heart. Within RV, we have detected statistically significantly increased expression of NPB in the diabetic rats compared to the controls. Based on our IF experiments, the source of NPB within RV are smooth muscle cells of the coronary circulation. Perfusion of the left ventricular and right ventricular myocardium differs during the cardiac cycle. Resting coronary blood flow in the RV occurs both in systole and diastole. Perfusion of RV is more vulnerable to changes in blood pressure than LV [36]. In diabetic patients, the function of RV is impaired [37,38] but the mechanism of its insufficient function has not yet been described. NPB could be involved in this process.

Cardiac spinal afferents convey nociceptive sensory information from the heart to the spinal cord throughout DRG, which are located in the nerve cell bodies of these sensory neurons [17]. These cardiac sensory fibers release different neuromediators, including calcitonin-gene-related peptide and/or substance P [8]. Results of our IF experiments demonstrated the presence of NPB in some of the neuronal bodies and nerve fibers but the absence of NPW in DRG. Additionally, some neurons express NPBWR1, as it is visible in the immunofluorescence pictures. Diabetes caused an increase in NPBWR1 mRNA expression but no protein expression difference was detected by the WB analysis. This can be explained by the fact that the total amount of NPBWR1 produced in DRG neurons is small and, at the same time, it is transferred to the neuronal fibers directed to the innervated organs and to the spinal cord. Therefore, no change in protein level was observed in the DRG itself. The involvement of the NPB/W signaling system in pain transmission has been already demonstrated in the partial sciatic nerve ligation model in rats [39]. The change in the expression level of NPBWR1 in DRG neurons caused by diabetes suggests the involvement of this signaling system in the pathophysiology of diabetic sensory neuropathy.

In conclusion, the NPB/W signaling system is involved in the regulation of heart functions and long-term diabetes leads to changes in the expression of individual members of this signaling system differently in each cardiac compartment, which is related to the different morphology and function of these cardiac chambers.

## 4. Materials and Methods

### 4.1. Experimental Animals

All these procedures were conducted following the National Institutes of Health “Guide for the Care and Use of Laboratory Animals” (NIH Publication No. 85-23, revised 1996), as well as the relevant Guidelines of the Czech Ministry of Agriculture for scientific experimentation on animals. All procedures and handling of the animals were reviewed by the University Committee for Experiments on Laboratory Animals. Approval for the experiments was granted by the Ministry of Education, Youth and Sports of the Czech Republic (MSMS-10669/2016-6; 15 March 2016).

Six-week-old male ZDF rats and lean Zucker rats (controls) were housed 1–2 per cage in a room at a constant temperature with a 12:12 h light–dark cycle. They were fed a diabetogenic diet Purina 5008 (Velaz, Prague, CZ) consisting of 56% carbohydrate, 27% protein, and 17% fat by kcal ad libitum with free access to drinking water. Body weights were measured once a week and blood glucose levels were measured twice per experiment (Table 1). Rats were sacrificed at 40 weeks of age by decapitation. The heart was rapidly excised, rinsed with ice-cold saline, freed of connective tissue and fat, and divided into the left atrium with the interatrial septum (LA), right atrium (RA), and free walls of left (LV) and right (RV) ventricles, and directly frozen in liquid N_2_ (for RNA and/or protein isolation), or embedded in optimum cutting temperature compound (Takara, San Jose, CA, USA) and frozen in precooled isopentane (for immunofluorescence). Stellate ganglia and upper thoracic DRG were excised and elaborated in the same way as the heart. Samples were kept at −80 °C until use. In total, sixteen animals were used for RNA isolation (eight ZDF and eight controls). For real time (RT)-qPCR, ten animals were for protein isolation (five ZDF and five controls) with subsequent western blotting, and six animals (three ZDF and three controls) for immunofluorescence.

### 4.2. RNA Isolation from Whole Tissues

Total RNA was isolated from each heart compartment (*n* = 8 of each) using TRI reagent (Sigma, St. Louis, MO, USA) following the protocol of the manufacturer. Contaminating DNA was destroyed with 1 U DNAse/μg of total RNA (Invitrogen, Carlsbad, CA, USA). Total RNA was isolated from DRG and stellate ganglia using RNasy Lipid Tissue Mini Kit (Qiagen, Hilden, Germany) according to the manufacturer’s protocol.

### 4.3. Laser Capture Microdissection (LCM)

Samples containing coronary arteries walls were obtained by LCM and subsequently used for RNA or protein isolation as described previously [40]. Cryostat sections of the left ventricle tissues from the control (*n* = 3) and ZDF (*n* = 3) rats were used for this experiment. Cryostat sections were stained with alum hematoxylin solution, washed with water, and dehydrated with ethanol. Coronary arteries were collected from 13-µm thick sections. For the RT-PCR analysis, RNA was isolated from obtained samples using RNeasy Micro Kits (Qiagen, Hilden, Germany) according to the manufacturer’s instructions. For the WB analysis, a 1.5 mm^2^ area was dissected for each sample and subsequently placed in 60 µL of laemmli buffer. The sample was heated for 10 min at 100 °C and stored at −20 °C. 

### 4.4. RT-qPCR Analysis

RNA obtained from whole tissues, as well as RNA obtained from LCM samples, was reverse-transcribed using Superscript III Reverse Transcriptase (Invitrogen, Carlsbad, CA, USA) for 50 min at 42 °C. Single-strand cDNA was synthesized from 3 μg of the total RNA. The RT-qPCR analysis was performed as described previously [16]. The primers were designed to amplify the sequence corresponding to nucleotides. Beta-actin (forward: TTCCTTCCTGGGTATGGAATC (821–841), reverse: GTTGGCATAGAGGTCTTTACGG (1008–1029); NPW (forward: GCTAGAGCCTTCGGTGAGAC (538–557), reverse: ATCGGTTCTTGAGACGGTCG (618–637); preproNPB (forward: GATGCGCCCAAGCGTAAGAA (207–226), reverse: TACACTGGAAAGTCCCTCGG (291–310); and NPBWR1 (forward: ACTCTAGTGTTGGGCTTCGC (625–644), reverse: CTAGCTGGATAGCACGCAGT (693–712).

Classical PCR reactions with subsequent agarose gel electrophoresis were first conducted to confirm the specificity of primers. Real-time qPCR was performed in the iCycler (Bio-Rad, Prague, Czech Republic). Final assay volumes were 15 μL and contained 7.5 μL iQ SYBR Green Supermix (Bio-Rad, Prague, Czech Republic), 0.15 μL of each primer (20 nmol/L), 3 μL of cDNA, and 4.2 μL of water. The quantitative PCR reactions were conducted as described previously [16]. Quantitative analysis of mRNAs of NPB, NPW, and NPBWR1 was performed in all heart chambers by subtracting their quantitative cycle values from (Cq) to Cq of reference gene. Β-actin was used as a reference gene based on its good expression stability. The relative expression ratios were calculated using the Livak (2^−ΔΔCT^) method.

### 4.5. Extraction of Protein

Rat heart tissues (*n* = 5–7 of each) were weighed and minced into small pieces. PBS along with a protease inhibitor cocktail was added to tissue in a ratio of 100 µL per 1 mg of tissue. Samples were homogenized for 3 min with a rest for 1 min in ice per cycle. Three cycles were used. Homogenate was centrifuged at 10,000× *g* for 15 min and debris was removed. The supernatant was collected and protein concentration was estimated by the Bradford protein assay. 

### 4.6. SDS-PAGE and the WB Analysis

Extracted proteins (25 µg/lane) were subjected to SDS-PAGE 12.5% under reducing conditions (2% *v*/*v* β-mercaptoethanol). The gel was transferred onto a nitrocellulose membrane (0.2 μm; Bio-Rad, Hercules, CA, USA) at 220 mA for 2 h at room temperature. The membrane was blocked with 5% milk powder in PBST (PBS pH 7.4, 0.1% TWEEN 20) for 1 h and incubated with anti-NPB (1:200; PA3-303, Thermo Fischer Scientific, Waltham, MA, USA), anti-NPW (1:1000; bs-11531R, Bioss Antibodies Inc, Woburn, MA, USA), and anti-NPBWR1 (1:2000; bs-8618R, Bioss Antibodies Inc, Woburn, MA, USA) for 1 h at room temperature or overnight at 4 °C. The membrane was washed three times (10 min per wash) with PBST and incubated with HRP-conjugated anti-IgGs (1:4000; A16116, Thermo Fisher Scientific, Waltham, MA, USA) for 45 min at room temperature. The membrane was washed three times as mentioned above with PBST. Detection was performed with a Westernbright ECL chemiluminescent reagent (Advansta, Menlo Park, CA, USA) and images were acquired using a ChemiDoc MP Imager (Bio-Rad, Hercules, CA, USA). Images were processed using the software package Imagelab 6.0.1 software (Bio-Rad, Hercules, CA, USA).

For the LCM-WB analysis, 20 µL of sample per lane was subjected to SDS-PAGE 12.5% gel, transferred onto the membrane, blocked for 1 h, and had an anti-NPBWR1 (1:2000) incubation overnight at 4 °C. A similar protocol was used as mentioned above with slight modifications, such as 1:2000 dilution of anti-IgGs for 45 min at room temperature. A similar blot was re-incubated with anti-ACTB (1:250; bs0061R; Bioss, Boston, MA, USA) for 1 h at room temperature. The membrane was washed three times (10 min per wash) with PBST and incubated with HRP-conjugated anti-IgGs (1:2000) for 45 min at room temperature. The membrane was washed three times as mentioned above with PBST. Detection was performed using a similar approach mentioned above.

### 4.7. Immunofluorescence

Shock-frozen hearts, stellate ganglia, and/or DRG (*n* = 3 of each) were cut into 10-μm thick sections using a Leica CM1850 cryostat (Leica, Bensheim, Germany) and fixed for 10 min in cold acetone. After 30 min of preincubation with normal goat serum (diluted 1:25 with PBS) at room temperature for 1 h, the sections were incubated with anti-NPB (1:200; PA3-303, Thermo Fischer Scientific, Waltham, MA, USA), anti-NPW (1:150; bs-11531R, Bioss Antibodies Inc, USA), or anti-NPBWR1 (1:200; bs-8618R, Bioss Antibodies Inc, Woburn, MA, USA) overnight at 4 °C. Subsequently, the samples were washed three times with PBS and incubated for 2 h at room temperature with FITC-conjugated anti-rabbit IgGs (1:400; F9887, Sigma, St. Louis, MO, USA). Preabsorption of primary antisera with appropriate peptide was performed in order to prove the specificity of binding as being already published [16]. Finally, sections were covered in DABCO mounting medium (Sigma, St. Louis, MO, USA) and observed with an Olympus BX 60 fluorescent microscope (Olympus, Prague, Czech Republic) equipped with appropriate filter combinations.

### 4.8. Ventricular Cardiomyocytes Isolation

The animals (the control rats *n* = 4 and the Zucker diabetic fatty rats with type 2 diabetes mellitus *n* = 4) were administered 1500 UI/kg b.w. heparin 15 min before sacrifice. After cervical dislocation, the heart was quickly removed, cannulated (aorta), washed with Ca^2+^-free Tyrode solution, and mounted to the constant-pressure Langendorff apparatus. The heart was perfused with warm (37 °C) and oxygenated solution. At the beginning with Ca^2+^-free Tyrode solution for 10 min, then with 0.6-μmol/L-Ca^2+^ Tyrode solution with 1.2 mg/mL of collagenase 2 (Worthington Biochemical Corporation, Lakewood, NJ, USA) and 1 mg/mL of bovine serum albumin (Sigma-Aldrich, St. Louis, MO, USA) for the next 13 min, followed by 0.6-μmol/L-Ca^2+^ Tyrode solution with 4 mg/mL of bovine serum albumin for the last 5 min. Cardiomyocytes were harvested from the left ventricle, filtered through 200 μm nylon mesh, washed, and stored in 0.2-mmol/L-Ca^2+^ Tyrode solution at room temperature.

### 4.9. Functional Measurement of Isolated Cardiomyocytes

Sarcometic contractions and calcium transients were measured as described previously [41]. The cardiomyocytes from the left ventricle (*n* = 16–23 in each group) were incubated with 2-μmol/L-Fura-2-AM Tyrode solution for 20 min and then washed with Tyrode solution repeatedly. The sarcomeric contractions and calcium transients were recorded in the Tyrode solution (control), 0.1-μmol/L-NPW Tyrode solution, and 0.1-μmol/L-NPB solution at 37 °C using the Ionoptix HyperSwitch Myocyte Calcium and Contractility System (IonOptix LLC, Westwood, CA, USA). The composition of the Tyrode solution was as follows (in mmol/L): NaCl 137, KCl 4.5, MgCl_2_ 1, CaCl_2_ 2, glucose 10, HEPES 5, and pH adjusted to 7.4 with NaOH. All chemicals were purchased from Sigma-Aldrich (St. Louis, MO, USA). During the measurement, the cells were stimulated with a field stimulator (MyoPacer Field Stimulator, IonOptix LLC, Westwood, CA, USA) at a frequency of 1 Hz. The offline analysis was performed with IonWizard 6.5 software (IonOptix LLC, Westwood, CA, USA).

### 4.10. Statistical Analysis

For qPCR and functional measurement of cardiomyocyte analysis, all the data are expressed as the mean ± standard error of the mean (SEM). Data obtained by qPCR from the individual experimental groups were first subjected to the Shapiro–Wilk normality test. As the results of this test showed that the values in the individual groups did not show a normal distribution, a nonparametric Mann–Whitney test was subsequently used for statistical evaluation. Values of *p* < 0.05 were considered statistically significant. The qPCR analysis was performed using the software package STATISTICA Cz, version 7 (StatSoft CR, Prague, Czech Republic). The results obtained from the functional measurement of isolated cardiomyocytes were statistically analyzed in OriginPro 2019 (OriginLab Corporation, Northampton, MA, USA).

For the WB analysis, each blot was first normalized by total protein lane and then normalized by either internal standard (a mixture of all tissues extracts) or a similar biological sample in the group to minimize blot-to-blot variation within the same group using similar antibodies and tissues, respectively. The mean of all the technical replicates (*n* = 3) or (*n* = 2) was used to analyze the expression of protein for each analyte (preproNPW in LA, NPWR1 in LV, NPWR1 in LA, and NPWR1 in DRG) except for preproNPB in RV. The chemiluminescence band value of each protein analyte in every animal was divided by the average intensity of the control group for this analyte (in other words, the average of the control group is set to 1).

In the WB analysis of preproNPB in RV, we observed a very low signal of preproNPB with a high background. To improve the signal of NPB, we increased the lysate amount, in some cases up to 50 µg per lane. Results obtained from these blots were first normalized with internal standards of previous blots (25 µg per lane) and corrected values of those blots were used for further analysis. Due to the high background, some technical replicates were also excluded from statistical calculation in the final WB analysis. We also considered some biological samples without technical replicate because excluding those samples were having negligible effects on the final results. All the data were expressed as the mean ± standard deviation. The difference in the expression between groups was calculated by a *t*-test. Moreover, the expression ratio was presented as an average ratio of signals from the diabetic to the control. Values of *p* < 0.05 were considered statistically significant changes in expression. 

## Figures and Tables

**Table 1:** Non-fasting plasma glucose levels and the body weight of the control and Zucker diabetic fatty (ZDF) rats with type 2 diabetes mellitus (*n* = 16 per group). Data are expressed in mean ± SD; *p*-value—ZDF vs. controls.

	Controls	ZDF	*p*-Value
Plasma glucose (mmol/L)	7.04 ± 1.39	30.21 ± 3.01	<0.0001
Body weight (g)	439.92 ± 25.97	416.58 ± 43.43	<0.09

## Data Availability

Not applicable.
